# Comoclathrin, a novel potent skin-whitening agent produced by endophytic *Comoclathris* strains associated with Andalusia desert plants

**DOI:** 10.1038/s41598-022-05448-9

**Published:** 2022-01-31

**Authors:** Katerina Georgousaki, Victor González-Menéndez, José R. Tormo, Nikolaos Tsafantakis, Thomas A. Mackenzie, Jesús Martín, Sentiljana Gumeni, Ioannis P. Trougakos, Fernando Reyes, Nikolas Fokialakis, Olga Genilloud

**Affiliations:** 1 Division of Pharmacognosy and Natural Products Chemistry, Department of Pharmacy, National and Kapodistrian University of Athens, Athens, Greece; 2 Fundación MEDINA, Health Sciences Technology Park, Granada, Spain; 3 Department of Cell Biology and Biophysics, Faculty of Biology, National and Kapodistrian University of Athens, Athens, Greece

**Keywords:** Chemical biology, Natural products, Structure elucidation, Metabolomics, Skin models

## Abstract

As part of our screening program for the discovery of molecules of microbial origin with skin-whitening activity, 142 diverse fungal endophytes from a wide variety of Andalusia arid plants were screened, applying the OSMAC approach. The fungal strains CF-090361 and CF-090766, isolated from xerophytic plants, were selected as the most promising, while phylogenetic analysis revealed that both strains could represent a new species within the genus *Comoclathris*. The effect of different fermentation conditions on the production of tyrosinase inhibitory activity was examined, in order to identify the optimum cultivation conditions. LCMS based metabolomics was applied to determine significant differences between the strains and fermentation conditions, and to identify potential bioactive secondary metabolites. Bioassay-guided purification of the main active components led to the isolation of three new compounds (**1–3**), along with the known compounds graphostrin B (**4**) and brevianamide M (**5**). Compound **1** (Comoclathrin) demonstrated the strongest anti-tyrosinase activity (IC_50_ 0.16 μΜ), which was 90-times higher than kojic acid (IC_50_ 14.07 μΜ) used as positive control. Additionally, comoclathrin showed no significant cytotoxicity against a panel of cancer cell lines (HepG2, A2058, A549, MCF-7 and MIA PaCa-2) and normal BJ fibroblasts. These properties render comoclathrin an excellent development candidate as whitening agent.

## Introduction

The term endophyte was first introduced by De Bary in 1866 and refers to an endosymbiotic group of microorganisms—including both bacteria and fungi—colonizing inter- and/or intracellular locations of plants, without causing any immediate pathogenic effect on its host(s)^1–4^. During this complex relationship with host plants endophytic fungi produce various biologically active secondary metabolites to increase their adaptability to both biotic and abiotic stresses^5^. Over the past two decades, endophytic fungi have attracted attention owing to their ability to produce many valuable compounds with high chemical diversity, including alkaloids, terpenoids, steroids, quinones, lignans, phenols and lactones^6,7^. Among them, several antimicrobial, immunosuppressant, insecticidal, cytotoxic, anticancer and antioxidant secondary metabolites have been reported^6,7^. However, and besides the wide range of bioactive substances produced by the endophytic microorganisms their potential to inhibit the adverse effects of tyrosinase enzyme has not been studied. Tyrosinase is the key enzyme of melanin biosynthesis in microorganisms, plants, and animals, playing an important role in the hydroxylation of L-tyrosine to L-3,4-dihydroxyphenylalanine (L-DOPA) and in the oxidation of L-DOPA to dopaquinone^8^. The only reported whitening agent isolated from endophytic fungi is kojic acid, which has been produced from strains of *Colletotrichum gloeosporioides* and *Aspergillus niger* isolated from *Sonneratia apetala* and from the roots of *Entandrophragma congoënse,* respectively^9,10^. Moreover, high levels of kojic acid are also produced by other endophytic fungal isolates belonging to the genera *Aspergillus*, *Petromyces*, *Penicillium*, *Chaetomium, Emericella and Pleospora,* and associated with common medicinal plants in Egypt^11^.

As part of our ongoing search for new tyrosinase inhibitors of microbial origin^12^, 142 diverse fungal endophytes from a wide variety of Andalusian arid plants were selected to be screened, applying the OSMAC (One Strain Many Compounds) approach by using four different fermentation media^13–16^. The two fungal strains CF-090361 and CF-090766, isolated from the xerophytic plants *Sedum sediforme* and *Nerium oleander*, were found to exhibit the most potent anti-tyrosinase effect when grown in LSFM and MMK2 media and were selected to be further studied. Their anti-tyrosinase activity was further confirmed when they were further tested in mouse melanocytes (B16F10 cell line) (see Supplementary material, Table S1).

This work describes the novelty of the producer strains as a new potential species of the genus *Comoclathris*, the identification of the best tyrosinase activity production conditions and the analysis of their metabolomic differences using volcano-plots based on Liquid Chromatography Mass Spectrometry (LCMS). The bioassay-guided isolation and structural elucidation of the main active components **1–5,** led to the identification of three new compounds (**1, 2, 3)** and the assessment of their in vitro whitening and cytotoxic activity.

## Results and discussion

### Phylogenetic analysis of the fungal endophytes

**Figure 1:**
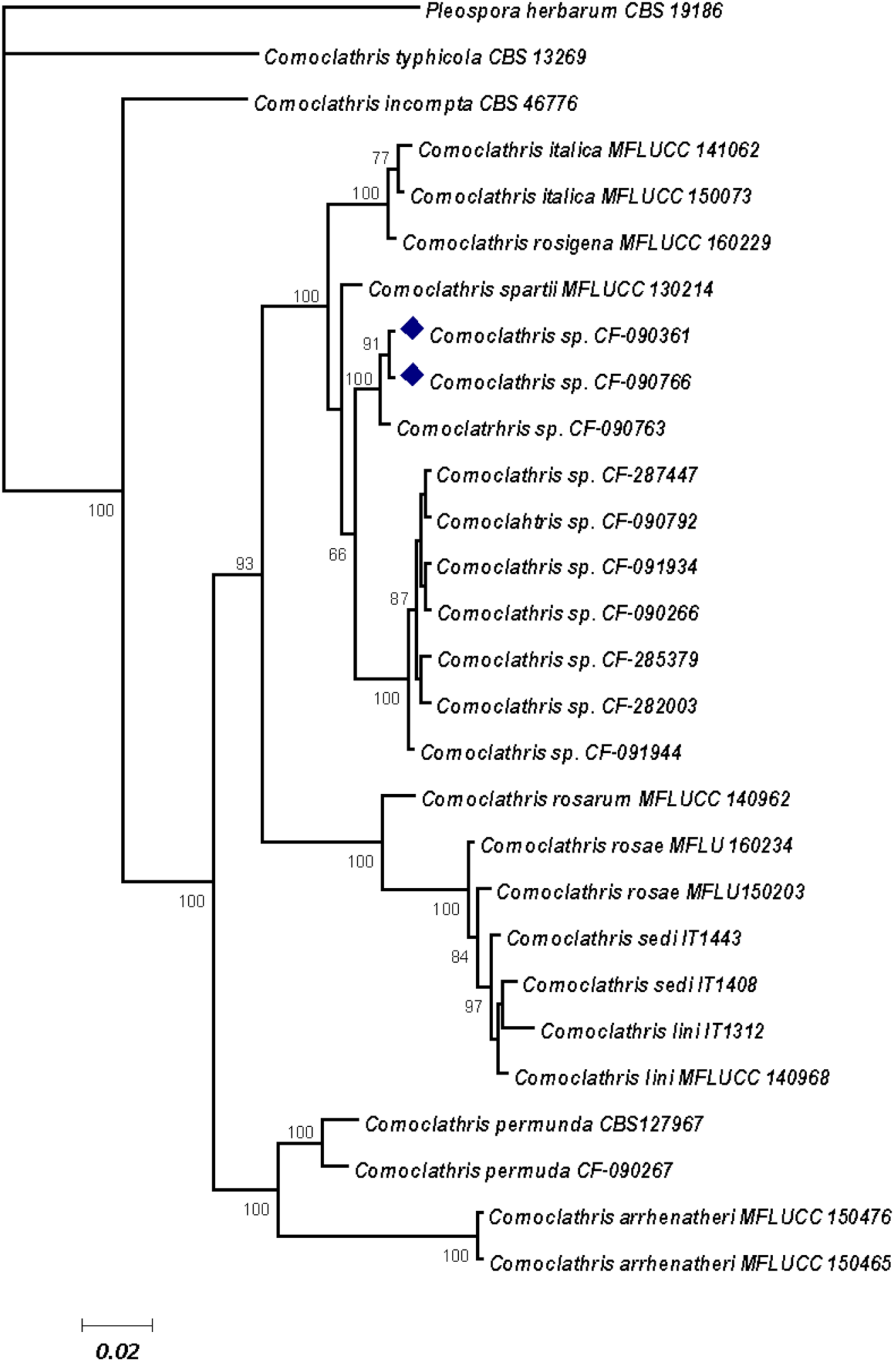
Bayesian inference of the ITS-28S sequence alignments of *Comoclathris* species. Clade probability values are indicated respectively on the branches.

The endophytic strains CF-090361 and CF-090766 were identified as potential new species of *Comoclathris* according to their ITS and 28S rDNA sequences and their phylogenetic position based on Bayesian inference (Fig. 1). The genus *Comoclathris* was introduced in 1909 (*Comoclathris* Clem., Gen. fung. (Minneapolis): 37 (1909)), and until now 44 species have been described [http://www.indexfungorum.org/names/Names.asp]. *Comoclathris* was originally wrongly associated with the genus *Alternaria*^17,18^ and it has been recently accommodated within the *Pleosporaceae* upon morphology and phylogenetic analysis. The consensus phylogenetic tree used in the study includes 11 *Comoclathris* endophytic strains isolated from arid plants of Andalusia and 17 GenBank™ sequences of representative *Comoclathris* species (see Table S1 in Supplementary information). However, additional multilocus phylogenetic analyses, morphological descriptions and their comparison with the recently added new *Comoclathris* species^19^ are still required to describe both endophytic strains as a new lineage within *Comoclathris*.

### Production conditions

Primary screening results had shown that the range of whitening activity in both strains was found to be dependent on the composition of the media used in the original OSMAC study (Table 1).

**Table 1:** Anti-tyrosinase activity of extracts from CF-090361 and CF-090766 cultivated in 4 fermentation media^20^ (Extracts were tested at a concentration corresponding to 0.02xWBE dilution (Whole Broth Equivalent)).

Strain	Fermentation medium	% Tyrosinase inhibitory effect
		(0.02xWBE)
CF-090766	LSFM	62.37 ± 3.35
	MMK2	23.66 ± 0.07
	XPMK	1.33 ± 2.48
	YES	− 7.52 ± 1.32
CF-090361	LSFM	73.52 ± 0.19
	MMK2	79.75 ± 0.63
	XPMK	11.51 ± 1.23
	YES	40.89 ± 0.61

The strongest anti-tyrosinase effect was observed when the strain CF-090361 was cultivated in the fermentation media LSFM and MKK2 and when the strain CF-090766 was cultivated in the medium LSFM for 14 days^16^. Parallel biological assays revealed the lack of any cytotoxic effect of the extracts against Hep-G2 and MCF-7 cancer cell lines. Both strains were then selected for further investigation using the fermentation medium LSFM. Common fermentation medium (i.e. LSFM) was chosen in order to compare the secondary metabolite production of the two strains.

Different culture conditions, with variations of cultivation parameters (i.e., incubation time and shape of culture format), were investigated, in order to define the optimum conditions for inducing the anti-tyrosinase effect of the two strains^14^. In order to investigate in a time course the production of tyrosinase inhibitory activity, the strains were grown in LSFM medium during 7, 14 and 21 days using as fermentation formats 40 mL EPA vials and 250 mL Erlenmeyer flasks, containing 10 and 50 mL of medium, respectively.

Evaluation of the anti-tyrosinase effect of the extracts showed that the best results were obtained for both strains when they were incubated for 7 days in flasks with high percentage of inhibition in the range of 80% (Table 2). This activity decreased rapidly after longer incubation of 14 and 21 days. In the case of the small volume vials, initial production at 7 days was drastically reduced when compared to the flask format (Table 2), and was only slightly improved after 21 days (data not shown). For this reason, extracts of ten replicates of the strains grown for 7 days in EPA vial and flask formats were regenerated and evaluated for their anti-tyrosinase activity (Table 2) and content in tyrosinase inhibitors using an untargeted metabolomic approach.

**Table 2:** Anti-tyrosinase activity of CF-090361 and CF-090766 extracts (7 days of incubation).

Strain	Fermentation format	Fermentation volume (mL)	% Tyrosinase inhibitory effect		
			(0.02xWBE)	(0.01xWBE)	(0.002xWBE)
CF-090361	EPA vial	10	33.30 ± 14.24	17.18 ± 7.38	11.30 ± 5.06
	250 mL FLASK	50	82.41 ± 0.83	81.64 ± 1.28	76.66 ± 3.42
CF-090766	EPA vial	10	56.59 ± 16.57	46.09 ± 18.73	20.08 ± 11.33
	250 mL FLASK	50	81.22 ± 0.49	82.85 ± 1.40	74.38 ± 4.64

*Each fermentation condition was performed in ten repetitions (n = 10).

### Untargeted metabolomics analysis

**Figure 2:**
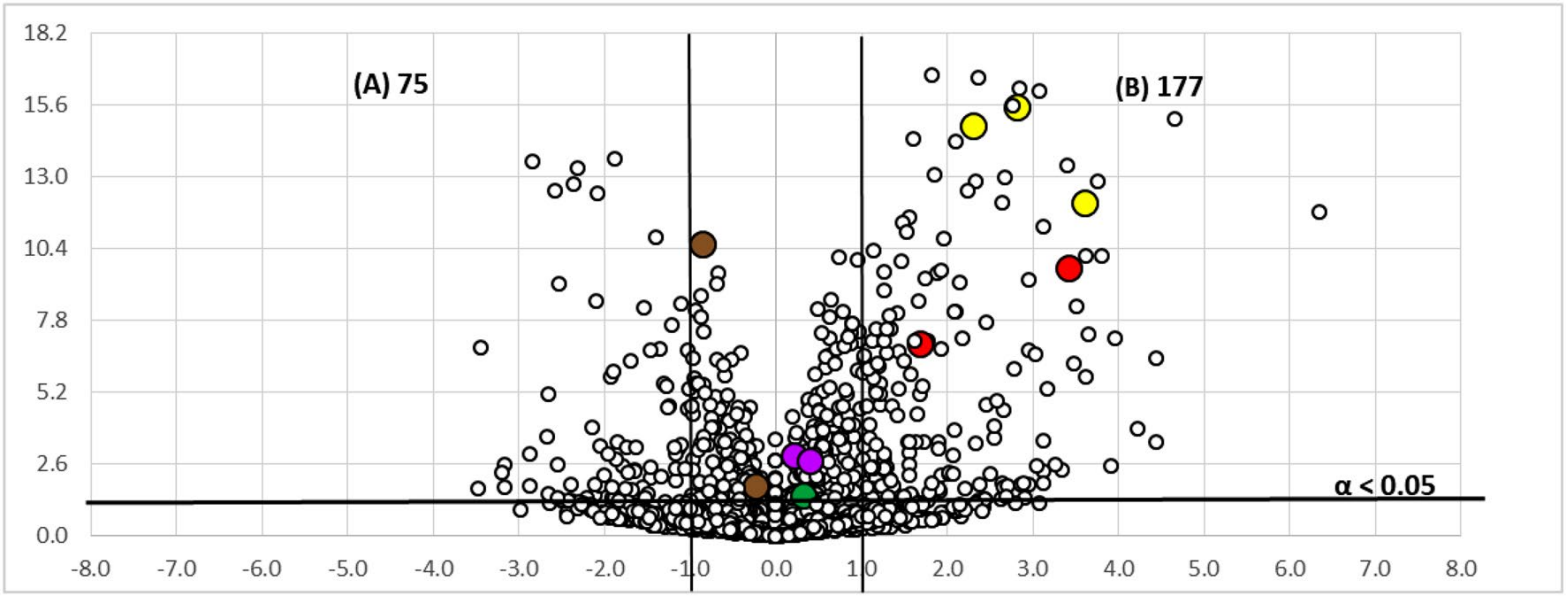
Volcano-plot statistical untargeted metabolomics comparison of the two fermentation formats tested for the strain CF-090361, comparing the different growth conditions (− log 10 of t-test statistical p-value in y-axis vs. − log 2 of ion masses areas ratio in x-axes): (**A**) culture in EPA vials; (**B**) culture in Flasks of 250 mL (*m/z* 219 and *m/z* 220 in red, *m/z* 205 and *m/z* 206 in purple, *m/z* 197 and *m/z* 198 in brown, *m/z* 223, *m/z* 241 and *m/z* 263 in yellow and *m/z* 322 in green). The number of statistically different mass ions due to higher production for each growth condition is indicated with statistical confidence of 95% (n = 10; α < 0.05) for each fermentation condition.

**Figure 3:**
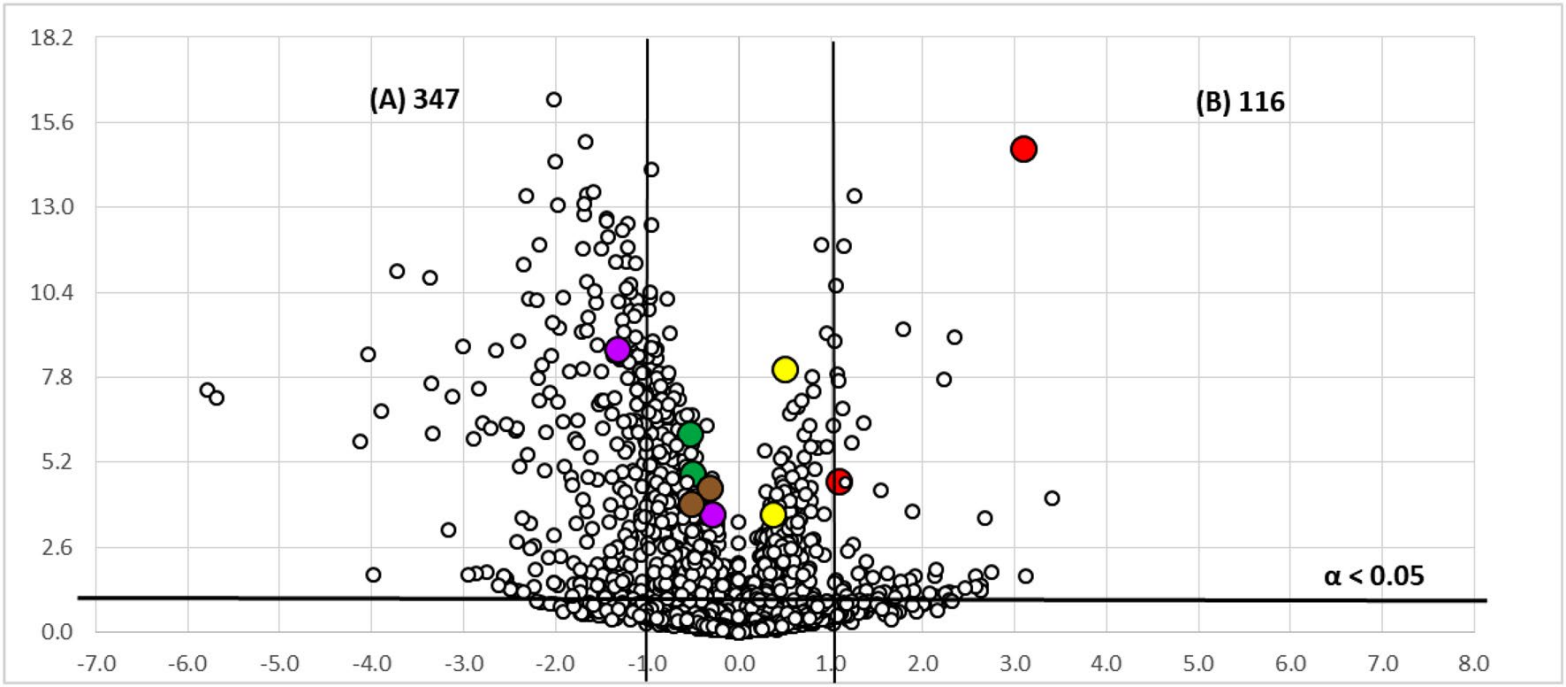
Volcano-plot statistical untargeted metabolomics comparison of the two fermentation formats tested for the strain CF-090766, comparing the different growth conditions (− log 10 of *t*-test statistical *p*-value in *y*-axis *vs*. − log 2 of ion masses areas ratio in *x*-axes): (**A**) culture in EPA vials; (**B**) culture in Flasks of 250 mL (*m/z* 219 and *m/z* 220 in red, *m/z* 205 and *m/z* 206 in purple, *m/z* 197 and *m/z* 198 in brown, *m/z* 223 and *m/z* 241 in yellow and *m/z* 304 and *m/z* 322 in green). The number of statistically different mass ions due to higher production for each growth condition is indicated with statistical confidence of 95% (*n* = 10; α < 0.05) for each fermentation condition.

Numerous studies have demonstrated that untargeted metabolomic studies using Volcano Plot analysis is a useful tool for the discovery of novel secondary metabolites produced differentially among conditions^21,22^. Volcano plots are scatter-plots used for the visualization of statistical results of omics data. In our case, they are used to visually describe how two different experimental conditions statistically affect a large set of components^23^. Thus, extracts generated from fermentations performed in ten replicates in both formats were analyzed by LC–MS and the metabolite differences between the two fermentation formats for each strain were visualized using Volcano Plots (Figs. 2, 3).

Volcano plots were generated by the comparison of the LR-MS profile of the extracts of the strains CF-090361 and CF-090766 grown in LSFM medium for 7 days in EPA vials and in 250 mL flasks. The points in the volcano plots that show a stronger combination of fold change and statistical significance in the active format, found in the upper-right or upper-left, represent the ions of the metabolites that are produced mainly in the active condition and are potential metabolites responsible for the activity of the extracts. On the other hand, the metabolites that have a relatively low fold-change between the two conditions, appear near the center.

The Volcano plots analyses indicated that a total of 177 and 116 ions are produced significantly (with more than > 95% of probability or α < 0.05) and in more than twofold increase in flasks than in EPA vials by the strains *Comoclathris* sp. CF-090361 and CF-090766, respectively.

Overexpressed ions in the active fermentation condition, as indicated by Volcano Plots, were used as a discrimination tool, in combination with the bioassay-guided isolation, for the fast identification of secondary metabolites exhibiting the most significant anti-tyrosinase activity.

### Bioassay-guided purification

For the isolation of the active components, the fermentation of the strains CF-090361 and CF-090766 was scaled-up to 1 L in LSFM medium and each strain was grown in flasks for 7 days, which are the selected optimum conditions. The purification of the active components was performed by parallel evaluation of the anti-tyrosinase activity and the LCMS profile of the fractions of the extracts of the two strains obtained by flash chromatography. The fractions that exhibited the most significant anti-tyrosinase activity were subjected to HRMS and LRMS analysis, in order to detect the pseudomolecular ions and fragment ions of interest, that were overexpressed in the active fermentation condition as indicated by Volcano Plots.

To this end, we found that the active fractions contained the ions at *m/z* 219 and 220 (in red colour) and at *m/z* 223, 241 and 263 (in yellow colour), were allocated in the upper-right part (flask) of the generated Volcano plots (Figs. 2, 3). In the case of the active extracts obtained from the fungal fermentation in flask format for both strains, the percentage of tyrosinase inhibitory activity was almost stable when the extracts were tested at various concentrations (0.02xWBE, 0.01xWBE and 0.002xWBE), while there was a significant decrease of the inhibitory activity in the case of the extracts obtained from the fermentation broths performed in EPA vials. This can be correlated for both strains with the presence of the compound with the ion at *m/z* 219 (highlighted in red) that it is produced in higher amounts in the flask format. Thus, our focus has been on the isolation, structure elucidation and biological evaluation of compounds containing this specific ion.

**Figure 4:**
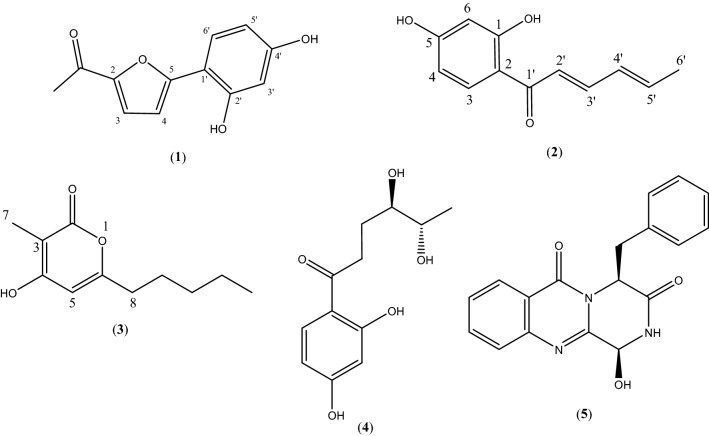
Chemical structures of compounds **1**–**5.**

Targeted chromatographic separations led to the isolation in both strains of one novel furan derivative, namely comoclathrin (**1**), one new sorbicillin analogue, namely 4,6-demethylsorbicillin (**2**) and one new violapyrone analogue, namely violapyrone L (VLP L) (**3**), together with two known compounds (**4** and **5**) (Fig. 4), isolated for the first time from the genus *Comoclathris*. Compounds **1**–**3** are reported as natural products for the first time, while compounds **2** and **3** have been previously mentioned as intermediate synthetic components^24,25^. Moreover compound **2** belongs to the group of “sorbicillinoids”, which are mainly produced by species of the fungal genus *Trichoderma*^26,27^. By comparing physical and spectroscopic data with literature values, the two known compounds were identified as the polyketide graphostrin B (**4**) and the indole alkaloid dimer brevianamide M (**5**), that has been isolated before from fungi belonging to the genus *Graphostroma* and *Aspergillus*, respectively^28,29^. To the best of our knowledge the genus *Comoclathris* has not been thoroughly studied regarding its chemical content, as only one compound, namely altersetin, has been isolated from strains belonging to this genus^30^.

### Chemical profile of the new purified natural products

Compound **1** gave a [M + H]^+^ ion at *m/z* 219.0651 in the (+)ESI-TOF MS appropriate for a molecular formula of C_12_H_10_O_4_ (calcd for C_12_H_11_O_4_, 219.0657) requiring 8 sites of unsaturation. Based on 1 and 2D NMR experiments (^13^C, HSQC-DEPT, HMBC), the 12 carbon atoms were assigned as five methines, one methyl and six quaternary carbons. The ^1^H NMR spectra of **1** (in DMSO-*d*_*6*_) showed characteristic signals of a 1,2,4-trisubstituted benzene ring at *δ*_H_ 7.57 [1H, d (*J* = 8.7 Hz), H-6’], 6.37 [1H, dd (*J* = 8.7, 2.4 Hz), H-5’] and 6.44 [1H, d (*J* = 2.4 Hz), H-3’], two olefinic protons at *δ*_H_ 7.46 [1H, d (*J* = 8.7 Hz), H-3] and 6.89 [1H, d (*J* = 8.7 Hz), H-4], attributed to a 2,5-disubstituted furan ring and one singlet at *δ*_H_ 2.42 (3H, s, H_3_-7) corresponding to the hydrogens of the acetyl group. Two additional singlets at *δ*_H_ 10.32 and 9.78 were identified, attributed to the exchangeable phenolic hydrogens at C-2’ and C-4’. The position of the hydroxy group at *δ*_H_ 10.32 was deduced by the HMBC correlations with the quaternary carbon at *δ*_C_ 108.34 (C-1’) and the methine C-3’ (*δ*_C_ 103.3). Likewise, the hydroxy group at *δ*_H_ 9.78 was assigned at the position C-4’ showing correlation peaks with C-5’ (*δ*_C_ 108.05) and C-3’. The cross peak between H-5’ and H-6’ in the ^1^H-^1^H COSY, their characteristic H–H coupling relationship (J = 8.7 Hz) of vicinal protons, and the heterocorrelation peaks with the quaternary carbons C-1’ and C-5 (*δ*_C_ 155.8) of the furan ring confirmed their relative position in the molecule. The presence of an additional methyl group at *δ*_H_ 2.42 (3H, s, H_3_-7) of the ^1^H NMR spectrum and its HMBC correlations with the carbonyl carbon 2-COCH_3_ (*δ*_C_ 185.1) and the aromatic oxygenated carbon of the furan ring at *δ*_C_ 149.7 (C-2) suggested the position of the acetyl group. Finally, the key HMBC correlations of C-2 and C-5 with methine protons H-3 and H-4 as well as their cross peak in the ^1^H-^1^H COSY spectra and coupling constant (^3^*J*_H/H_) of 8.7 Hz confirmed the presence of the 2,5-disubstituted furan ring. According to the above evidence, structure of compound **1** was elucidated as 1-(5-(2,4-dihydroxyphenyl) furan-2-yl) ethan-1-one and given the trivial name comoclathrin.

**Figure 5:**
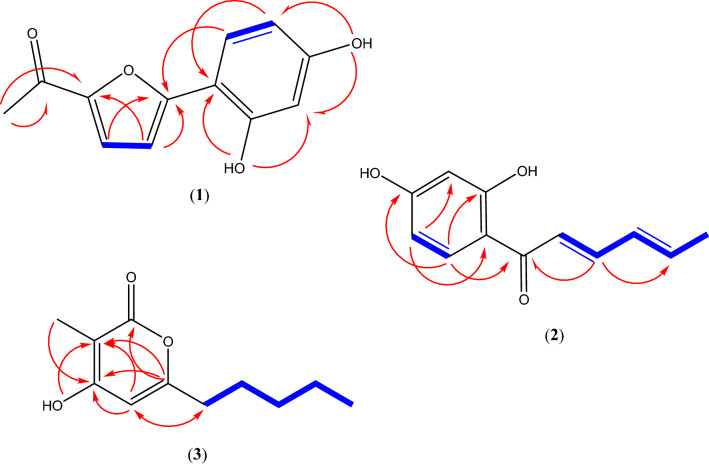
Key HMBC (H → C) and ^1^H-^1^H COSY (bold lines) correlations of compounds **1** − **3.**

Compound **2** gave a [M + H]^+^ ion at *m/z* 205.0869 in the (+)ESI-TOF MS, appropriate for a molecular formula of C_12_H_12_O_3_ (calcd for C_12_H_13_O_3_, 205.0865) requiring 7 sites of unsaturation. The ^1^H NMR spectrum (in CD_3_OD) displayed characteristic signals of a 1,2,4-trisubstituted benzene ring at *δ*_H_ 7.82 [1H, d (*J* = 8.9 Hz), H-3], 6.38 [1H, dd (*J* = 8.9, 2.3 Hz), H-4] and 6.27 [1H, d (*J* = 2.3 Hz), H-6], of a conjugated *trans* double bond system at *δ*_H_ 7.11 [1H, d (*J* = 14.8 Hz), H-2’], *δ*_H_ 7.42 [1H, dd (*J* = 14.8, 10.9 Hz), H-3’], *δ*_H_ 6.44 [1H, dd (*J* = 14.7, 10.9 Hz), H-4’] and *δ*_H_ 6.33 [1H, m, H-5’], and of a methyl group at *δ*_H_ 1.90 [3H, d (*J* = 6.4 Hz), H_3_-6’]. Analysis of the ^13^C NMR spectrum revealed twelve signals. Based on an HSQC-DEPT experiment, the aforementioned signals were assigned as seven methines and one methyl carbon. The remaining four signals in the ^13^C NMR spectrum were deduced as quaternary, corresponding to the carbonyl carbon C-1’ at *δ*_C_ 192.4, to the oxygenated aromatic carbons C-1 and C-5 at *δ*_C_ 165.1 and 167.0, and to the quaternary carbon C-2 (*δ*_C_ 113.2). Comparison of HRMS, ^1^H- and ^13^C-NMR spectra (Table 3) with literature data suggested compound **2** as a polyketide analogue of sorbicillin and 6-demethylsorbicillin, showing a common sorbyl group but with no aromatic methyl protons^26,31^. This hypothesis was further confirmed by 2D NMR (Fig. 5). In fact, the key ^1^H-^1^H COSY correlations between H-2’/H-3’, H-3’/H-4’, H-4’/H-5’, H-5’/ H_3_-6’ and the long-range HMBC heterocorrelations from H-3’ to C-1’ and C-5’, from H-3 to C-1’, C-5 and C-1 and from H-4 to C-6 and C-2 unambiguously assigned the relative position of all protons and carbons. Consequently, compound **2** was identified as a new sorbicillin analogue, namely 4,6-demethylsorbicillin.

**Table 3:** ^1^H (500.13 MHz) and ^13^C NMR (125.76 MHz) data of Compounds **1–3.**

Position	1^a^		2^b^		3^a^	
	δ_C_, type	δ_H_ (J in Hz)	δ_C_, type	δ_H_ (J in Hz)	δ_C_, type	δ_H_ (J in Hz)
1			165.1, C			
2	149.7, C		113.2, C		165.06, C	
3	121.7, CH	7.46, d (8.7)	133.3, CH	7.82, (d, 8.9)	96.3, C	
4	109.5, CH	6.89, d (8.7)	109.2, CH	6.38, dd (2.4/8.9)	165.17, C	
5	155.8, C		167.0, C		99.6, CH	5.98, s
6			103.8, CH	6.27, d (2.3)	163.2, C	
7					8.7, CH_3_	1.74, brs
8					32.9, CH_2_	2.40, t (7.5)
9					26.3, CH_2_	1.53, qui (7.5)
10					22.1, CH_2_	1.27, m
11					30.8, CH_2_	1.29, m
12					14.1, CH_3_	0.88, t (7.1)
1′	108.3, C		192.4, C			
2′	156.9, C		123.2, CH	7.11, dd (14.8)		
3′	103.3, CH	6.44, d (2.4)	145.7, CH	7.42, dd, (10.9/14.8)		
4′	159.9, C		131.9, CH	6.44, dd, (14.7/10.9)		
5′	108.1, CH	6.37,dd (8.7, 2.4)	142.1, CH	6.33, m		
6′	127.7, CH	7.57, d (8.7)	18.9, CH_3_	1.90, d (6.4)		
2-CO-CH_3_	185.1, C					
2-CO-CH_3_	26.1, CH_3_	2.42, brs				
4-OH						11.14, brs
2′-OH		10.32, brs				
4′-OH		9.78, brs				
6′-OH			167.5			

^a^Data obtained in DMSO-*d*6.

^b^Data obtained in MeOD.

Compound **3** gave a [M + H]^+^ ion at *m/z* 197.1191 in the (+)-ESI-TOF MS, accounting for a molecular formula of C_11_H_16_O_3_ (calcd for C_11_H_17_O_3_ 197.1177) requiring 4 sites of unsaturation. Analysis of chemical shifts, peak multiplicities and peak areas of the ^1^H NMR spectrum suggested characteristic signals of a linear C-5 alkyl chain at *δ*_H_ 2.40 [2H, t (7.5), H_2_-8], 1.53 [2H, quint, H_2_-9], 1.29 [2H, m, H_2_-10], 1.27 [2H, m, H_2_-11], 0.88 [3H, t (7.1), H_3_-12], an olefinic methine proton at *δ*_H_ 5.98 (1H, s, H-5), and a methyl group at *δ*_H_ 1.74 [3H, s, H_3_-7]. One additional singlet at *δ*_H_ 11.14 was identified, attributed to the exchangeable hydroxy group at C-4. The hypothesis of a linear penta-alkyl chain was further confirmed by 2D NMR. Analysis of the ^1^H-^1^H COSY experiment suggested a proton spin system from H-8 to H-12 while the HSQC-DEPT experiments unambiguously assigned relative carbons (C-8 to C-12) as four methylenes at *δ*_C_ 32.9 (C-8), *δ*_C_ 26.3 (C-9), *δ*_C_ 22.1 (C-10), *δ*_C_ 30.8 (C-11) and one methyl carbon at *δ*_C_ 14.1 (C-12). The relative position of the olefinic proton H-5 and of the methyl group at *δ*_H_ 1.74 (H_3_-7) was established by the common HMBC correlation with the quaternary carbon C-3 at *δ*_C_ 96.3 and by the key correlation of the hydroxy proton at C-4 (*δ*_C_ 165.17) with C-3 and C-5 (*δ*_C_ 99.6). The ^13^C-NMR revealed one more signal of oxygenated carbon (*δ*_C_ 165.06) which was attributed to C-2 of the compound. Further HMBC correlations of the methyl group at *δ*_H_ 1.74 with the oxygenated carbons C-2 and C-4 together with the λ_max_ at 289.0 and the fact that compound **3** needs to form a ring to satisfy the unsaturation number indicated the presence of a typical α-pyrone chromophore. The α-pyrone ring was identified as 3-methyl-4-hydroxypyran-2-one with a linear pentyl moiety at C-6 established by the HMBC correlation of H-8 with the methine carbon C-5. Thus, the structure of **3** was determined as 4-hydroxy-3-methyl-6-pentyl-2H-pyran-2-one and represents a new violapyrone analogue, named violapyrone L (VLP L)^32–34^.

Compounds **4** and **5** were identified as graphostrin B and brevianamide M respectively, based on NMR and HRMS data, and by comparison with those reported in the literature^28,29^.

### Activity profiling of purified natural products

Purified compounds **1**–**5** were evaluated for their tyrosinase inhibitory activity using mushroom tyrosinase. The highest anti-tyrosinase activity was presented by compounds **1**, **2** and **5,** with IC_50_ values ranging from 0.16 to 6.81 μΜ (see Supplementary material, Table S2). Compound **1** (comoclathrin), exhibited the most potent activity, showing an 87*-*times higher inhibitory effect (IC_50_ = 0.16 μΜ) as compared to the positive control kojic acid (IC_50_ = 14.07 μΜ). On the other hand, compounds **3** and **4** did not demonstrate a significant inhibitory effect (IC_50_ > 100 μΜ) (Table S2).

To evaluate the cytotoxicity and possible specificity of compounds **1**–**5**, five different cancer cell lines were chosen (HepG2 as a cytotoxic indicator, and A2058, A549, MCF-7 and MIA PaCa-2 as complementary cell lines to study potential selectivity). Compounds **2** and **5** showed slight cytotoxic activity against A2058 (90.00 uΜ) and HepG2 (25.00 uΜ), while compound **1,** having the most potent anti-tyrosinase effect, did not present activity against any of the cancer cell lines tested (see Supplementary material, Table S2). Comoclathrin (compound **1**) was also found to exert no cytotoxicity on normal human dermal fibroblast cell line, which suggests that this compound could be safely used for its whitening activity.

**Figure 6:**
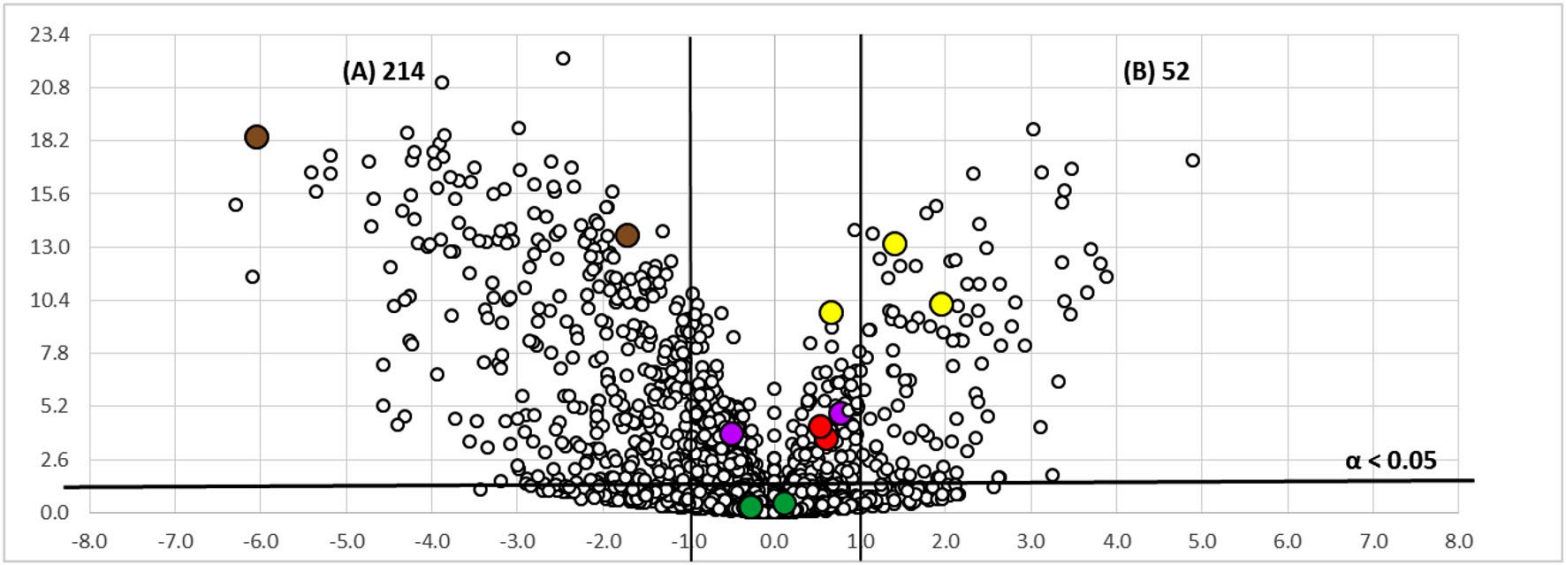
Volcano-plot statistical untargeted metabolomics comparison of the strain CF-090766 and CF-090361 cultured for 7 days in the same fermentation format (flasks of 250 mL), comparing the metabolite production by the two strains (− log 10 of *t*-test statistical *p*-value in *y*-axis *vs*. − log 2 of ion masses areas ratio in *x*-axes): strain CF-090766; strain CF-090361 (compound **1** in red, compound **2** in purple, compound **3** in brown, compound **4** in yellow and compound **5** in green). The number of statistically different mass ions due to higher production for each growth condition is indicated with statistical confidence of 95% (n = 10; α < 0.05) for each fermentation condition.

Volcano plots presented in Figs. 2 and 3 show visually how the different fermentation conditions affect the production of active components. Our results confirm that volcano plots may be used as a complementary tool for the fast identification of bioactive secondary metabolites in the frame of a bio-guided isolation process. In fact, it is noteworthy to mention that the ion *m/z* 219, which was overexpressed in the active culture conditions in both strains and found abundant in several active fractions of the extracts of both strains (Figs. 2, 3), corresponds to the protonated adduct of the active compound **1**, the one with the highest anti-tyrosinase activity. Furthermore, volcano plot analysis was successfully applied to describe how the two different strains grown in the same conditions differ regarding the identified set of active metabolites (Fig. 6). The active compounds **1**, **2** and **5** appear to be produced at the same level by both strains CF-090361 and CF-090766, as their ions are detected near the center of the volcano plot. On the other hand, compound **3** (ions highlighted in brown) production is 64-fold higher in the strain CF-090766 than in the strain CF-09036, while compound** 4** (ions highlighted in yellow) is produced 4 times more by the strain CF-090361.

In conclusion, our approach was successfully applied to the fungal endophytic *Comoclathris* strains CF-090766 and CF-090361, highlighting the optimum conditions for the production of metabolites with anti-tyrosinase activity. Bioassay-guided isolation led to the identification of the three new compounds (compounds **1–3)**, which demonstrated a high anti-tyrosinase activity without showing toxicity against a panel of cancer and normal cell lines. Furthermore, our findings highlighted that conspecific strains of the genus *Comoclathris*, isolated from different plants and geographic areas, demonstrated a relevant whitening effect by producing the same active metabolites. More importantly the furan derivative comoclathrin (**1**) has been shown to be a potent tyrosinase inhibitor devoid of cytotoxic effect with excellent properties and development potential as whitening agent.

## Methods

### General experimental procedure

^1^H and ^13^C NMR spectra were obtained at 500 MHz, using a Bruker Avance III 500 MHz spectrometer (500 MHz and 125 MHz for ^1^H and ^13^C NMR respectively) equipped with a low volume 1.7 mm inverse detection microcryoprobe. HRESIMS and LC-UV–MS data were measured using a Bruker maXis QTOF mass spectrometer coupled to an Agilent 1200 HPLC system and on an Agilent 1100 single quadrupole LC–MS system, as previously described^35^. Preparative HPLC was performed on a Gilson 322 System using a Xbridge™ C18 (19 × 250 mm, 5 μm) column at a flow rate of 14 mL/min. Semipreparative HPLC was performed on the same system using a Xbridge™ C18 (10 × 150 mm, 5 μm) column or a Xbridge Prep Phenyl, (10 × 150 mm, 5 μm) column at a flowrate of 3.6 mL/min. Evaporation of solvents was performed on a vacuum rotary evaporator (Rotavapor R-3000r, Buchi, Postfach, Switzerland). The acetone employed for extraction, as well as the solvents used for isolation were of analytical and HPLC grade, respectively.

### Strain isolation and characterization

The endophytic fungi CF-090361 and CF-090766 were isolated by using standard indirect isolation techniques from stems of *Sedum sediforme* (S.Alhamilla, Almeria, Spain) and *Nerium oleander* (Tabernas, Almeria, Spain), respectively^16^. Frozen stock cultures in 10% glycerol (− 80 °C) are maintained in the culture collection of Fundación MEDINA. DNA extraction, PCR amplification, DNA sequencing and Bayesian phylogenetic analysis were performed following an already described process^36^. Sequences of the complete ITS_1_-5.8S-ITS_2_-28S region or independent ITS and partial 28S rDNA sequences were compared with sequences at GenBank®, the NITE Biological Resource Center (http://www.nbrc.nite.go.jp) and CBS strain database (http://www.westerdijkinstitute.nl) by using the BLAST® application. Species affinities of *Comoclathris* was inferred from Bayesian analysis using the Markov Chain Monte Carlo (MCMC) approach with MrBayes 3.01^37^. Akaike Information Criterion (AIC) of the nucleotide substitution models was calculated using MrModeltest® 2.2 software^38^, being GTR + I + G was the selected model for the alignment.

### Small scale extraction for the screening of the fungal endophytes

142 diverse fungal strains were revived from cryotubes containing fungal mycelia discs in 10% (v/v) glycerol following a procedure as described by González-Menéndez^20^. In order to evaluate the impact of different nutritional conditions (OSMAC approach) on the rate of secondary metabolites production by this fungal strain, four different fermentation media (LSFM, MMK2, XPMK and YES)^20^ were used for the liquid-state cultures. These four media formulations were selected based on their reported ability to induce the production of high chemical diversity in taxonomically diverse fungal strains^20^. The cultures and the small scale extractions were performed by following an already described procedure^20^.

### Fungal fermentation and extraction for the untargeted metabolomics study

The fungal strains were revived from cryotubes containing fungal mycelia discs in 10% (v/v) glycerol following a procedure as described by González-Menéndez^20^. After 7 days of incubation at 22 °C, two sets of fermentations were performed and the strains were inoculated in the culture medium LSFM^20^, where they exhibited the most significant tyrosinase inhibitory activity, as indicated during the screening process.

The first set of fermentations was performed in duplicate in vials (40 mL EPA vials) and 250 mL Erlenmeyer flasks containing 10 and 50 mL of medium respectively. Fungal inocula cultured in SMYA medium during 7 days at 22 °C, 220 rpm and 70% relative humidity were used to inoculate EPA vials and Flasks at 3% of final volume (v/v) following previously described protocols^20,39^. All fermentation broths were incubated for 7, 14 and 21 days, in order to investigate the best time course of the production of the bioactive metabolites. Biological assay revealed that 7 days is the optimum time of fungal incubation.

The second set of ten repetitions of each fermentation condition was performed also in 40 mL EPA vials and in 250 mL flasks containing 10 mL and 50 mL of LSFM medium respectively, for 7 day, as this was the optimum time of the fungal incubation. All fermentation broths were extracted with acetone, and samples were finally prepared in a final 20% dimethyl sulfoxide (DMSO)/ water solution at two whole broth equivalent (WBE) concentration according to the procedure previously described^20^.

### HPLC–UV-LRMS profile analysis, metabolomics and quantification

The culture extracts (2 µL) were analyzed by HPLC–UV-LRMS. LC analysis was performed on an Agilent 1200 (Santa Clara, CA, USA), using a Zorbax SB-C8 column (2.1 × 30 mm, 1.8 µm) with guard column, maintained at 40 °C with a flow rate of 300 µL/min and 210 nm UV detection. Mass spectrometry acquisition was performed on an Agilent MSD 1100 mass low resolution spectrometer to generate the metabolomic raw data. The solvents and gradient system used, as well as the statistical analyses by t-test and metabolomic charts (volcano-plots) were performed by following an already described process^40^.

### Scale up fungal fermentation, extraction and fractionation

1 L scale up fermentation of both strains CF-090361 and CF-090766 was performed by inoculating aliquots of 1.5 mL of the each inoculum into twenty 250 mL flasks containing 50 ml of LSFM medium obtained following previously described protocol^20^. Inoculated flasks were incubated during seven days at 22 °C, 220 rpm and 70% relative humidity in a shaking incubator (Kühner AG,Birsfelden,Suiza).

The scale up fermentation broths (1 L) were extracted with acetone (1 L) under continuous shaking at 220 rpm for 1 h and centrifugation was followed. The remaining mixture (ca. 2 L) was concentrated to ca. 1 L under a nitrogen flow. The solution was loaded, with continuous 1:1 water dilution, keeping the flow-through on a column packed with SP-207ss reversed-phase resin (brominated styrenic polymer, 65 g) previously equilibrated with water. The loaded column was further washed with water (2 L). For the extract of the strain CF-090361, the elution was performed at 10 mL min^−1^ on an automatic flash chromatography system (CombiFlash Rf, Teledyne Isco), using a linear gradient from 5 to 20% acetone in water (in 15.8 min) with a final 100% acetone step (for 19.2 min), collecting 35 fractions of 20 mL. For the extract of the strain CF-090766 the elution was performed at 18 mL min^−1^ on an automatic flash chromatography system (CombiFlash Rf, Teledyne Isco), using an isocratic at 5% acetone in water (for 6 min), followed by an isocratic at 20% acetone in water (for 6 min), an isocratic at 40% acetone in water (for 6 min), an isocratic at 60% acetone in water (for 6 min), an isocratic at 80% acetone in water (for 6 min), and an isocratic at 100% acetone (for 20 min), collecting 48 fractions of 20 mL. Fractions were concentrated to dryness on a centrifugal evaporator, tested for their tyrosinase inhibitory activity and forwarded for further chemical investigation.

### Bioassay-guided purification of the active metabolites

Compound **1** (1 mg) was obtained from fraction F24 of CF-090766 using a Xbridge Prep Phenyl, (10 × 150 mm, 5 μm) column and a gradient method as follows: 0–20 min, 40% MeOH (solvent B) (isocratic); 20–40 min 100% B (linear gradient); 40–45 min, 100% B (isocratic); 45–47 min, 40% B (linear gradient). Flow rate was set at 3.6 ml/min, detection wavelength at 210 and 350 nm and the retention time was 11.8 min.

Compound **2** (0.5 mg) was obtained from fraction F30 of CF-090766 using a ReproSil 100 C18, (250 cm × 10 mm, 5 μm) column and a gradient method as follows: 0–18 min, 40 to 47% CH_3_CN (solvent C) (isocratic); 23–28 min 100% C (linear gradient). Detection wavelengths were set at 316 and 346 nm and the retention time was 19.23 min.

Compound **3** (0.9 mg) was obtained from fraction F26 of CF-090766 using a Xbridge™ C18 (10 × 150 mm, 5 μm) column and a gradient method varying as follows: 0–2 min, 20% C (isocratic); 32–35 min 60% C (linear gradient); 40–50 min, 100% B (linear gradient). Flow rate was set at 3.6 ml/min, detection wavelength was set at 254 nm and the retention time of the fraction containing compound **3** was 18 min. Then compound **3** was further purified by using the same column and the following gradient method: 0–5 min, 20% B (isocratic), 10–30 min, 30% (linear gradient), 35–40 min, 40% B (linear gradient), 50–55 min, 100% B (linear gradient). Detection wavelength was set at 210 nm and the retention time of the pure compound **3** was 23 min.

Compound **4** (2.7 mg) was obtained from fraction F19 of CF-090361 using a Xbridge™ C18 (19 × 250 mm, 5 μm) column and a gradient method as follows: 0–5 min, 5% C (isocratic); 20–25 min 100% C (linear gradient). Flow rate was set at 14 ml/min, detection wavelength was set at 254 nm and the retention time was 15.85 min.

Compound **5** (0.2 mg) was obtained from fraction F21 of CF-090361 using a Xbridge™ C18 (10 × 150 mm, 5 μm) column and a gradient method varying as follows: 0–2 min, 2% C (isocratic); 40–45 min 100% C (linear gradient). Flow rate was set at 3.6 ml/min, detection wavelength was set at 210 nm and the retention time of the fraction containing compound **5** was 21.5 min. Then compound **5** was further purified by using the same column and the following gradient method: 0–5 min, 50% B (isocratic), 10–20 min, 70% (linear gradient), 30–35 min, 100% B (linear gradient). Detection wavelength was set at 210 nm and the retention time of the pure compound **5** was 15 min.

1-(5-(2,4-dihydroxyphenyl)furan-2-yl)ethan-1-one (**1**): brownish solid; UV (MeOH) λmax 210 nm and 350 nm; ^1^H NMR and ^13^C NMR data see Table 1; HRMS (ESI-Q-TOF) *m/z*: [M + H] ^+^ calcd for C_12_H_11_O_4_ 219.0657; found 219.0651.

(2E,4E)-1-(2,4-dihydroxyphenyl)hexa-2,4-dien-1-one (**2**): yellow powder; UV (MeOH) λmax 316 nm and 346 nm; ^1^H NMR and ^13^C NMR data see Table 1; HRMS (ESI-Q-TOF) *m/z*: [M + H] ^+^ calcd for C_12_H_13_O_3_ 205.0865; found 205.0869.

4-hydroxy-3-methyl-6-pentyl-2H-pyran-2-one (**3**): brown solid; UV (MeOH) λmax 316 nm and 346 nm; ^1^H NMR and ^13^C NMR data see Table 1; HRMS (ESI-Q-TOF) *m/z*: [M + H] ^+^ calcd for C_11_H_17_O_3_ 197.1177; found 197.1191.

Graphostrin B (**4**): white powder; ^1^H NMR and ^13^C NMR data were consistent with those previously reported^29^; HRMS (ESI-Q-TOF) *m/z*: [M + H] ^+^ calcd for C_12_H_17_O_5_ 241.1076; found 241.1068.

Brevianamide M (**5**): colorless crystals; ^1^H NMR and ^13^C NMR data were consistent with those previously reported^28^; HRMS (ESI-Q-TOF) *m/z*: [M + H]^+^ calcd for C_18_H_16_N_3_O_3_ 322.1191; found 322.1183.

### Enzymatic tyrosinase inhibitory activity

The effects of the isolated compounds **1**–**5** on the diphenolase activities of mushroom tyrosinase were investigated by evaluating their capacity to inhibit the catalytic action of tyrosinase in the oxidation of L-DOPA to dopachrome. An already described method using mushroom tyrosinase, a lyophilized powder, ≥ 1000 units/mg solid (Sigma-Aldrich, EC Number: 1.14.18.1), was employed^41^. Kojic acid (5 to 30 μM) and potassium buffer were used as a positive and negative controls, respectively. All experiments were performed in triplicate.

### Cell-based anti-tyrosinase activity

During the screening process, the anti-tyrosinase effect of the extracts was also evaluated on mouse skin melanoma cells (B16-F10) as previously described^12^. Analysis were performed in triplicates and the initial crude extracts were tested at 0.01xWBE dilution. **Cytotoxic evaluation.** The cytotoxic activity of compounds **1**–**5** was measured through the MTT (3-(4,5-dimethyl-2-thiazolyl)-2,5-diphenyl-2H-tetrazolium bromide) assay against HepG2, A2058, A549, MCF-7 and MIA PaCa-2 cell lines, following an already described process^42–44^. Doxorubicin (8 point dose–response curve with 1/3 dilutions starting at 50 μM) and DMSO (0.5%) were used as a positive and negative controls, respectively. All experiments were performed in triplicate.

Moreover, the effect of the active compound **1** on the viability of the human foreskin fibroblasts (BJ), frequently used for testing of skin active natural compounds, obtained from the American Tissue Culture Collection (ATTC), was examined, using the MTT method, at concentrations 0.5 μΜ, 1 μΜ, 2 μΜ, 3 μΜ, 4 μΜ and 5 μΜ, using an already described process^45^.

## Supplementary Information

Supplementary Information 1.

## Abbreviations

L-DOPA: L-3,4-dihydroxyphenylalanine
OSMAC: One strain many compounds
LCMS: Liquid chromatography–mass spectrometry
WBE: Whole broth equivalents
HRMS: High resolution mass spectrometry
LRMS: Low resolution mass spectrometry
NMR: Nuclear magnetic resonance
HMBC: Heteronuclear multiple bond correlation
COSY: Homonuclear correlation spectroscopy
CD_3_OD: Deuterated methanol
HSQC: Heteronuclear single quantum coherence
DNA: Deoxyribonucleic acid
PCR: Polymerase chain reaction
DMSO: Dimethyl sulfoxide
