# Supplementary material for: Comoclathrin, a novel potent skin-whitening agent produced by endophytic Comoclathris strains associated with Andalusia desert plants
Source: Sci Rep. 2022 Jan 31;12:1649. doi: 10.1038/s41598-022-05448-9 (PMC8803924; doi:10.1038/s41598-022-05448-9)
Supplement: Supplementary file 1 — Supplementary Information 1. [file 41598_2022_5448_MOESM1_ESM.doc]

**Comoclathrin, a novel potent skin-whitening agent produced by endophytic *Comoclathris* strains associated with Andalusia desert plants.**

Katerina Georgousaki^1,2^, Victor González-Menéndez^2^, José R. Tormo^2^, Nikolaos Tsafantakis^1^, Thomas A. Mackenzie^2^, Jesús Martín^2^, Sentiljana Gumeni^3^, Ioannis P. Trougakos^3^, Fernando Reyes^2^, Nikolas Fokialakis^1^*^*^* and Olga Genilloud^2*^

^1^ *Division of Pharmacognosy and Natural Products Chemistry, Department of Pharmacy, National and Kapodistrian University of Athens, Athens, Greece*

^2^ *Fundación MEDINA, Health Sciences Technology Park, Granada, Spain*

*^3^ Department of Cell Biology and Biophysics, Faculty of Biology, National and Kapodistrian University of Athens, Athens, Greece*

^*^ Authors of correspondence: [fokialakis@pharm.uoa.gr](mailto:fokialakis@pharm.uoa.gr); [olga.genilloud@medinaandalucia.es](mailto:olga.genilloud@medinaandalucia.es)

Table of Contents

[**Figure S1:** Skin whitening activity of the extracts of the strains CF-090361 and CF-090766.. 4](#__RefHeading___Toc87303542)

[**Table S1:** Fungal strains included in the phylogenetic analysis 5](#__RefHeading___Toc87303543)

[**Figure S2.** ^1^H NMR spectrum of compound 1 (DMSO-*d*6) 6](#__RefHeading___Toc87303544)

[**Figure S3**. ^1^H -^1^H COSY NMR spectrum of compound **1** (DMSO-*d*6) 6](#__RefHeading___Toc87303545)

[**Figure S4**. HSQC NMR spectrum of compound **1** (DMSO-*d*6) 7](#__RefHeading___Toc87303546)

[**Figure S5**. HMBC NMR spectrum of compound **1** (DMSO-*d*6) 7](#__RefHeading___Toc87303547)

[**Figure S6**. ^13^C NMR spectrum of compound **1** (DMSO-*d*6) 8](#__RefHeading___Toc87303548)

[**Figure S7**. ESI(+)-HRMS spectrum of compound **1** 8](#__RefHeading___Toc87303549)

[**Figure S8**. UV spectrum of compound **1** in MeOH 9](#__RefHeading___Toc87303550)

[**Figure S9.** ^1^H NMR spectrum of compound **2** (MeOD) 9](#__RefHeading___Toc87303551)

[**Figure S10**. ^1^H -^1^H COSY NMR spectrum of compound **2** (MeOD) 10](#__RefHeading___Toc87303552)

[**Figure S11**. HSQC NMR spectrum of compound **2** (MeOD) 10](#__RefHeading___Toc87303553)

[**Figure S12**. HMBC NMR spectrum of compound **2** (MeOD) 11](#__RefHeading___Toc87303554)

[**Figure S13**. ^13^C NMR spectrum of compound **2** (MeOD) 11](#__RefHeading___Toc87303555)

[**Figure S14**. ESI(+)-HRMS spectrum of compound **2** 12](#__RefHeading___Toc87303556)

[**Figure S15**. UV spectrum of compound **2** in MeOH 12](#__RefHeading___Toc87303557)

[**Figure S16.** ^1^H NMR spectrum of compound **3** (DMSO-*d*6) 13](#__RefHeading___Toc87303558)

[**Figure S17**. ^1^H -^1^H COSY NMR spectrum of compound **3** (DMSO-*d*6) 13](#__RefHeading___Toc87303559)

[**Figure S18**. HSQC NMR spectrum of compound **3** (DMSO-*d*6) 14](#__RefHeading___Toc87303560)

[**Figure S19**. HMBC NMR spectrum of compound **3** (DMSO-*d*6) 14](#__RefHeading___Toc87303561)

[**Figure S20**. ^13^C NMR spectrum of compound **3** (DMSO-*d*6) 15](#__RefHeading___Toc87303562)

[**Figure S21**. ESI(+)-HRMS spectrum of compound **3** 15](#__RefHeading___Toc87303563)

[**Figure S22**. UV spectrum of compound **3** in MeOH 16](#__RefHeading___Toc87303564)

[**Table S2:** In vitro tyrosinase inhibitory activity and cytotoxicity of the isolated compounds 1-5. 16](#__RefHeading___Toc87303565)

[**Figure S23:** Relative (%) cell survival (MTT assay) of BJ fibroblasts exposed to the indicated concentrations of the compound **1** for 24 h. 16](#__RefHeading___Toc87303566)


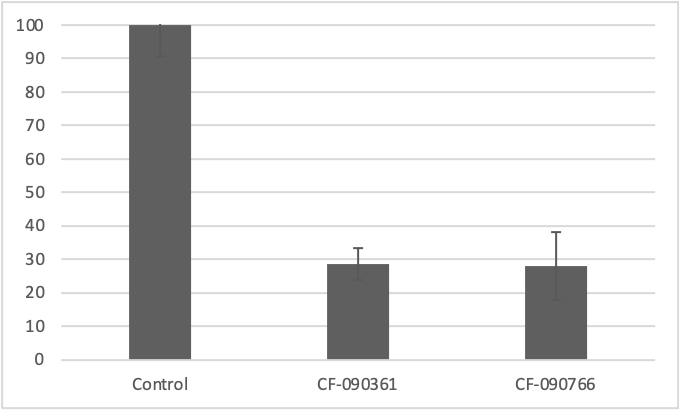


# **Figure S1:** Skin whitening activity of the extracts of the strains CF-090361 and CF-090766. Relative (%) tyrosinase activity in mouse melanocytes after 24h of treatment with the micro-fractions at 0.01xWBE dilution. Bars, SD (n3); *P < 0.05. Controls (cells treated with the vehicle/DMSO) were set to 100%.

# **Table S1:** Fungal strains included in the phylogenetic analysis (**strains** **isolated** from plants collected in arid zones of Andalucía are **in bold**).

| **Species** | **Strain code ^a^** | **Substrate** | **Origin** | **Genbank accesion numbers^b^** | |
| --- | --- | --- | --- | --- | --- |
|  |  |  |  | **ITS** | **28S** |
| *Pleospora herbarum var herbarum* | CBS 191.86 | *Medicago sativa* | India | KC584239 | JX681120 |
| *Comoclathris typhicola* | CBS 132.69 | *Typha angustifolia* | Netherlands | MH859275 JF740325 | |
| *Comoclathris incompta* | CBS 467.76 | *Olea europaea* | Greece | KY940770 GU238087 | |
| *Comoclathris italica* | MFLUCC 14-1062 | *Vicia sp.* | Italy | KY771326 KY771322 | |
| *Comoclathris italica* (ex-type) | MFLUCC 15-0073 | *Thalictrum* sp. | Italy | KX500109 KX500105 | |
| *Comoclathris rosigena* (ex-type) | MFLUCC 16-0229 | *Rosa canina* | Italy | MG828879 MG828995 | |
| *Comoclathris spartii* | MFLUCC 13-0214 | *Spartium junceum* | Italy | KM577159 | KM577160 |
| ***Comoclathris* sp** | **CF-090361** | ***Sedum sediforme*** | **Sierra Alhamilla, Almeria, Spain** | MG065810 | |
| ***Comoclathris* sp** | **CF-090763** | ***Nerium oleander*** | **Tabernas desert, Almeria, Spain** | MG065811 | |
| ***Comoclathris* sp.** | **CF-287447** | ***Rosmarinus eriocalyx*** | **Tabernas desert, Almeria, Spain** | MG065804 | |
| ***Comoclathris* sp.** | **CF-090792** | ***Lygeum spartum*** | **Tabernas desert, Almeria, Spain** | MG065805 | |
| ***Comoclathris* sp.** | **CF-091934** | ***Lygeum spartum*** | **Tabernas desert, Almeria, Spain** | MG065806 | |
| ***Comoclathris* sp.** | **CF-090266** | ***Lygeum spartum*** | **Tabernas desert, Almeria, Spain** | MG065803 | |
| ***Comoclathris* sp.** | **CF-285379** | ***Fagonia cretica*** | **Tabernas desert, Almeria, Spain** | MG065808 | |
| ***Comoclathris* sp** | **CF-282003** | ***Retama sphaerocarpa*** | **Albuñuelas, Granada, Spain** | MG065809 | |
| ***Comoclathris* sp.** | **CF-091944** | ***Launaea arboresceus*** | **Tabernas desert, Almeria, Spain** | MG065807 | |
| *Comoclathris rosarum* (ex-type) | MFLUCC 14-0962 | *Rosa canina* | Italy | NR_157507 | MG828994 |
| *Comoclathris rosae* | MFLUCC 16-0234 | *Rosa canina* | Italy | MG828877 MG828993 | |
| *Comoclathris rosae* (ex-type) | MFLUCC 15-0203 | *Rosa canina* | Italy | MG828876 MG828992 | |
| *Comoclathris sedi* | IT1443 = MFLUCC 14-0761 | *Digitalis* sp. | Italy | KP334718 KP334708 | |
| *Comoclathris sedi* | IT1408= MFLUCC 13-0763 | *Rosa sp.* | Italy | KP334717 KP334707 | |
| *Comoclathris lini* (ex-type) | IT1312= MFLUCC 14-0561 | *Ononis spinosa* | Italy | KT591615 KT591615 | |
| *Comoclathris lini* (ex-type) | MFLUCC 14-0968 | *Linum sp.* | Italy | KR049218 | KR049219 |
| *Comoclathris permunda* | CBS 127967 |  | USA | MH864779 | MH876217 |
| ***Comoclathris permunda*** | **CF-090267** | ***Lygeum spartum*** | **Tabernas desert, Almeria, Spain** | MG065813 | |
| *Comoclathris arrhenatheri* | MFLUCC 15-0476 | *Dactylis glomerata* | Italy | KY026595 | KY000648 |
| *Comoclathris arrhenatheri (*ex-type) | MFLUCC 15-0465 | *Arrhenatherum elatius* | **I**taly | KX965737 KY000647 | |

^a^ CBS, CBS-KNAW Fungal Biodiversity Centre, Utrecht, the Netherlands; CF, Fundación MEDINA Private Fungal Collection, Granada, Spain; MFLUCC, Mae Fah Luang University Culture Collection, Chiang Rai, Thailand;

^b^ 28S, large subunit of the nrDNA; ITS, internal transcribed spacer regions of the nrDNA and intervening 5.8S nrDNA

# **Figure S2.** ^1^H NMR spectrum of compound 1 (DMSO-*d*6)

# **Figure S3**. ^1^H -^1^H COSY NMR spectrum of compound **1** (DMSO-*d*6)

# **Figure S4**. HSQC NMR spectrum of compound **1** (DMSO-*d*6)

# **Figure S5**. HMBC NMR spectrum of compound **1** (DMSO-*d*6)

# **Figure S6**. ^13^C NMR spectrum of compound **1** (DMSO-*d*6)


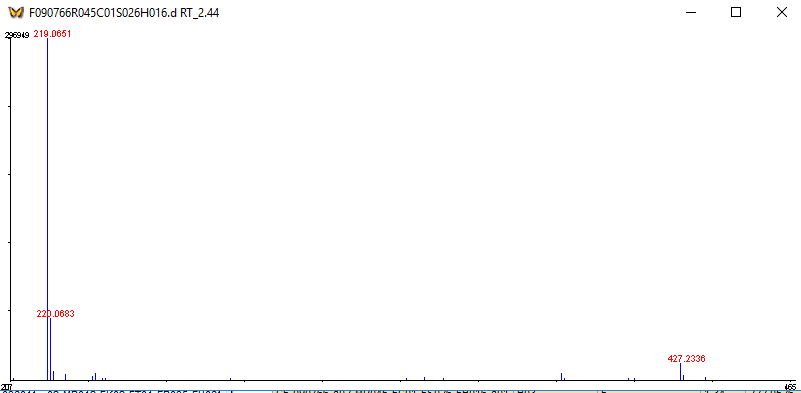


# **Figure S7**. ESI(+)-HRMS spectrum of compound **1**


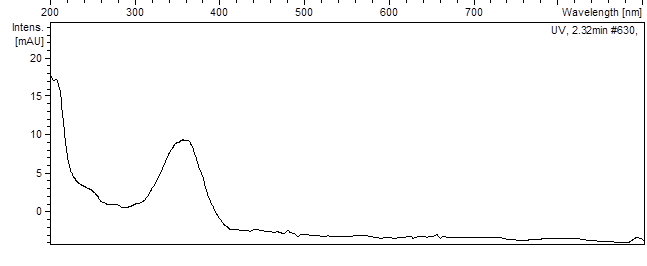


# **Figure S8**. UV spectrum of compound **1** in MeOH

# **Figure S9.** ^1^H NMR spectrum of compound **2** (MeOD)


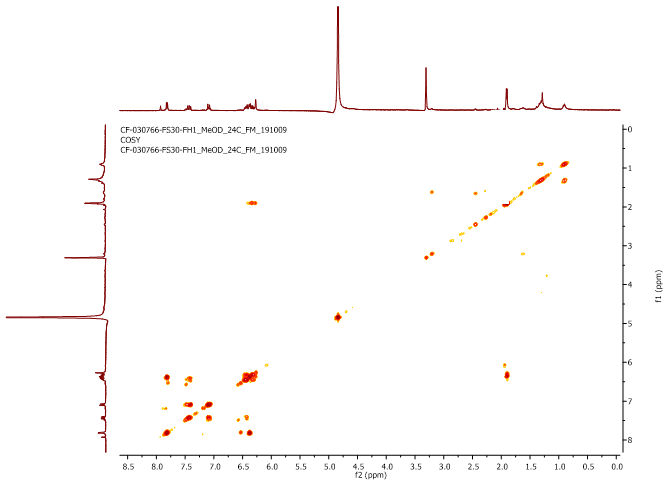


# **Figure S10**. ^1^H -^1^H COSY NMR spectrum of compound **2** (MeOD)


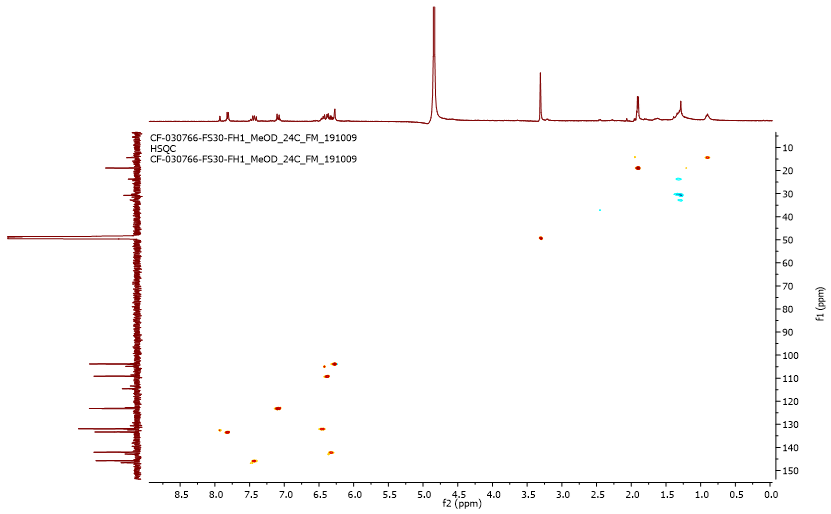


# **Figure S11**. HSQC NMR spectrum of compound **2** (MeOD)


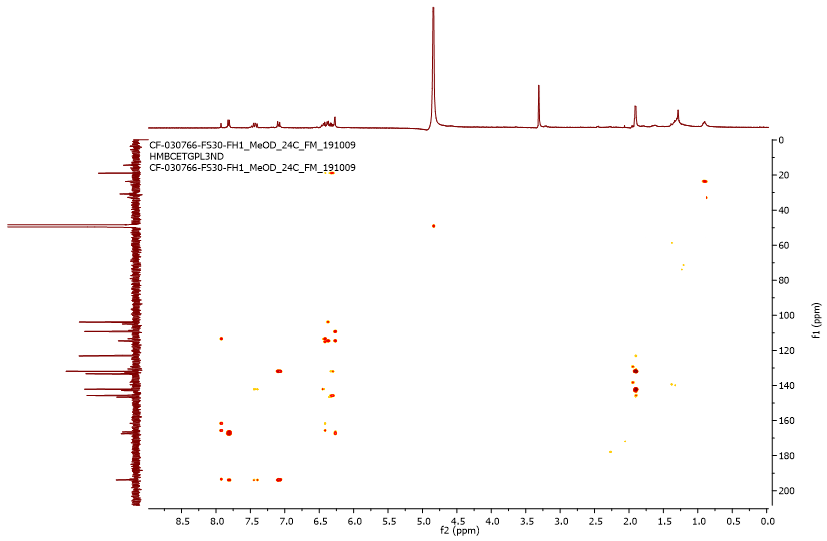


# **Figure S12**. HMBC NMR spectrum of compound **2** (MeOD)

# **Figure S13**. ^13^C NMR spectrum of compound **2** (MeOD)


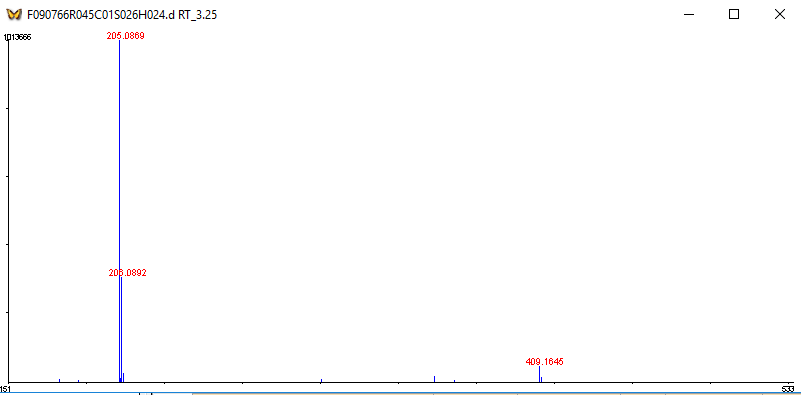


# **Figure S14**. ESI(+)-HRMS spectrum of compound **2**


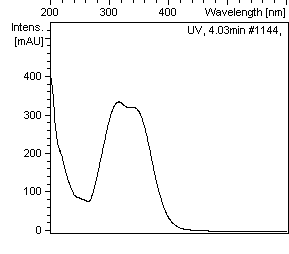


# **Figure S15**. UV spectrum of compound **2** in MeOH

# **Figure S16.** ^1^H NMR spectrum of compound **3** (DMSO-*d*6)

# **Figure S17**. ^1^H -^1^H COSY NMR spectrum of compound **3** (DMSO-*d*6)

# **Figure S18**. HSQC NMR spectrum of compound **3** (DMSO-*d*6)

# **Figure S19**. HMBC NMR spectrum of compound **3** (DMSO-*d*6)

# **Figure S20**. ^13^C NMR spectrum of compound **3** (DMSO-*d*6)


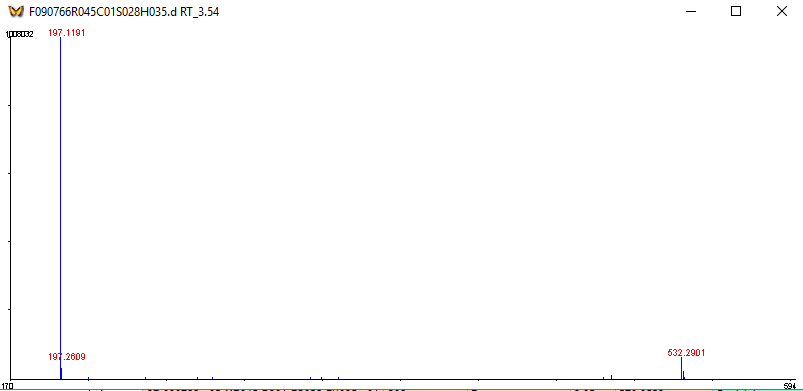


# **Figure S21**. ESI(+)-HRMS spectrum of compound **3**


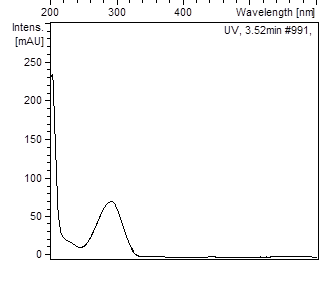


# **Figure S22**. UV spectrum of compound **3** in MeOH

# **Table S2: In vitro tyrosinase inhibitory activity and cytotoxicity of the isolated compounds 1-5.**

| **Compound** | **Tyrosinase inhibitory effect** | **Cytotoxic effect** | | | | |
| --- | --- | --- | --- | --- | --- | --- |
|  |  | HepG2 | A2058 | A549 | MCF-7 | MIA PaCa-2 |
|  | ^a^IC_50_ values | ^b^ED_50_ values | | | | |
| **1** | 0.16 | > 100 | > 100 | > 100 | > 100 | > 100 |
| **2** | 3.5 | > 100 | 90 | > 100 | > 100 | > 100 |
| **3** | >100 | > 100 | > 100 | > 100 | > 100 | > 100 |
| **4** | >100 | > 100 | > 100 | > 100 | > 100 | > 100 |
| **5** | 6.81 | 25 | > 100 | > 100 | > 100 | > 100 |
| **(+) Control** | 14.07^c^ | < 0.11^d^ | < 0.11^d^ | 3.76^d^ | 1.52^d^ | 1.02^d^ |

^a^IC_50_ and ^b^ED_50_ in μM for compounds **1**-**5** and controls (+): ^c^Kojic acid; ^d^Doxorubicin


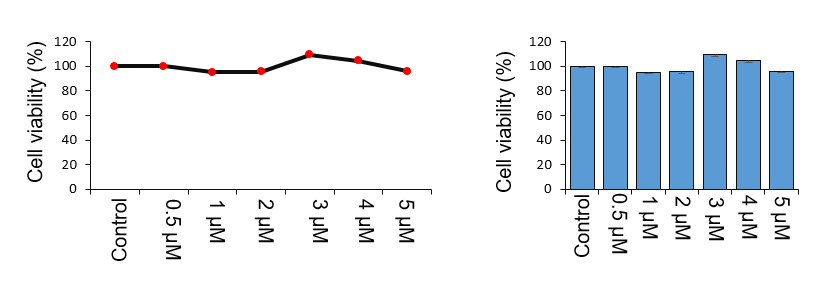


# **Figure S23:** Relative (%) cell survival (MTT assay) of BJ fibroblasts exposed to the indicated concentrations of the compound **1** for 24 h.
